# Supplementary material for: Impact of COVID-19 on hospital admission of acute stroke patients in Bangladesh
Source: PLoS One. 2021 Jan 13;16(1):e0240385. doi: 10.1371/journal.pone.0240385 (PMC7806171; doi:10.1371/journal.pone.0240385)
Supplement: S1 Data — (DOCX) [file pone.0240385.s001.docx]

| **Pre-Covid** | | | | | | | | **Covid** | | | | | | | |
| --- | --- | --- | --- | --- | --- | --- | --- | --- | --- | --- | --- | --- | --- | --- | --- |
| **No. of Week** | **Week** |  | **IST** | **ICH** | **SAH** | **Others** | **Total** | **No. of Week** | **Week** |  | **IST** | **ICH** | **SAH** | **Others** | **Total** |
| **1.** | Jan 1-Jan 7 | Male | 8 | 12 | 0 | 0 | 20 | **13.** | Mar 25 - Mar 31 | Male | 7 | 21 | 0 | 0 | 28 |
|  |  | Female | 1 | 10 | 7 | 0 | 18 |  |  | Female | 2 | 4 | 2 | 0 | 8 |
|  |  | Total |  |  |  |  | 38 |  |  | Total |  |  |  |  | 36 |
| **2.** | Jan 8- Jan 14 | Male | 8 | 14 | 1 | 0 | 23 | **14.** | April 1 - April 7 | Male | 5 | 21 | 2 | 0 | 28 |
|  |  | Female | 4 | 21 | 3 | 0 | 28 |  |  | Female | 5 | 18 | 4 | 0 | 27 |
|  |  | Total |  |  |  |  | 51 |  |  | Total |  |  |  |  | 55 |
| **3.** | Jan 15-Jan 21 | Male | 11 | 16 | 1 | 2 | 30 | **15.** | April 8 - April 14 | Male | 0 | 0 | 0 | 0 | 0 |
|  |  | Female | 2 | 12 | 4 | 0 | 18 |  |  | Female | 0 | 2 | 1 | 0 | 3 |
|  |  | Total |  |  |  |  | 48 |  |  | Total |  |  |  |  | 3 |
| **4.** | Jan 22- Jan 28 | Male | 4 | 17 | 1 | 1 | 23 | **16.** | April 15-April 21 | Male | 1 | 1 | 0 | 0 | 2 |
|  |  | Female | 5 | 11 | 5 | 1 | 22 |  |  | Female | 0 | 3 | 1 | 0 | 4 |
|  |  | Total |  |  |  |  | 45 |  |  | Total |  |  |  |  | 6 |
| **5.** | Jan 29- Feb 4 | Male | 8 | 16 | 3 | 0 | 27 | **17.** | April 22 - May 28 | Male | 6 | 19 | 3 | 1 | 29 |
|  |  | Female | 4 | 14 | 4 | 0 | 22 |  |  | Female | 6 | 15 | 1 | 0 | 22 |
|  |  | Total |  |  |  |  | 49 |  |  | Total |  |  |  |  | 51 |
| **6.** | Feb 5- Feb 11 | Male | 4 | 11 | 2 | 0 | 17 | **18.** | April 29 - May 05 | Male | 7 | 16 | 2 | 0 | 25 |
|  |  | Female | 5 | 9 | 6 | 0 | 20 |  |  | Female | 8 | 16 | 3 | 0 | 27 |
|  |  | Total |  |  |  |  | 37 |  |  | Total |  |  |  |  | 52 |
| **7.** | Feb 12- Feb 18 | Male | 14 | 18 | 4 | 1 | 37 | **19.** | May 6 - May 13 | Male | 7 | 14 | 2 | 1 | 24 |
|  |  | Female | 7 | 15 | 4 | 0 | 26 |  |  | Female | 6 | 11 | 3 | 1 | 21 |
|  |  | Total |  |  |  |  | 63 |  |  | Total |  |  |  |  | 45 |
| **8.** | Feb 19 - Feb 25 | Male | 9 | 23 | 5 | 1 | 38 | **20.** | May 13 - May 19 | Male | 7 | 1 | 0 | 0 | 8 |
|  |  | Female | 3 | 12 | 10 | 0 | 25 |  |  | Female | 3 | 7 | 0 | 0 | 10 |
|  |  | Total |  |  |  |  | 63 |  |  | Total |  |  |  |  | 18 |
| **9.** | Feb 26 - Mar 3 | Male | 4 | 20 | 1 | 0 | 25 | **21.** | May 20-May 26 | Male | 8 | 11 | 4 | 1 | 24 |
|  |  | Female | 8 | 20 | 9 | 1 | 38 |  |  | Female | 3 | 4 | 1 | 1 | 9 |
|  |  | Total |  |  |  |  | 63 |  |  | Total |  |  |  |  | 33 |
| **10.** | Mar 4 - Mar 10 | Male | 4 | 17 | 7 | 0 | 28 | **22.** | May 27-June 2 | Male | 8 | 12 | 3 | 0 | 23 |
|  |  | Female | 12 | 24 | 5 | 0 | 41 |  |  | Female | 2 | 6 | 3 | 0 | 11 |
|  |  | Total |  |  |  |  | 69 |  |  | Total |  |  |  |  | 34 |
| **11.** | Mar 11 - Mar 17 | Male | 13 | 19 | 5 | 0 | 37 | **23.** | June 3 - June 9 | Male | 5 | 16 | 1 | 0 | 22 |
|  |  | Female | 5 | 22 | 5 | 0 | 32 |  |  | Female | 7 | 6 | 2 | 0 | 15 |
|  |  | Total |  |  |  |  | 69 |  |  | Total |  |  |  |  | 37 |
| **12.** | Mar 18 - Mar 24 | Male | 5 | 16 | 3 | 0 | 24 | **24.** | June 10 – June 16 | Male | 6 | 16 | 0 | 0 | 22 |
|  |  | Female | 5 | 12 | 3 | 0 | 20 |  |  | Female | 7 | 10 | 3 | 2 | 22 |
|  |  | Total |  |  |  |  | 44 |  |  | Total |  |  |  |  | 44 |

*IST= Ischemic Stroke, ICH= Intracerebral Hemorrhage, SAH= Subarachnoid Hemorrhage
