# Supplementary material for: Impact of COVID-19 on hospital admission of acute stroke patients in Bangladesh
Source: PLoS One. 2021 Jan 13;16(1):e0240385. doi: 10.1371/journal.pone.0240385 (PMC7806171; doi:10.1371/journal.pone.0240385)
Supplement: Attachment — Submitted filename: Response to Reviewers.docx [file pone.0240385.s002.docx]

**Response to Reviewers**

**Reviewer #1:**

Comment 1: Need to have the manuscript reviewed by a science/technical writer would be useful as the discussion section has a lot of verbiage.

Authors’ response: We thank the reviewer for his/her advice. We have now rewritten the discussion section to remove the verbiage as much as possible and have the manuscript reviewed by a professional technical writer.

Comment 2: There are typographical errors which can be addressed by spell check

Authors’ response: We apologize for overlooking the errors. We have now corrected the typographical errors we have identified.

Reviewer #2:

Comments: The manuscript requires some grammatical assistance.
The overall information regarding stroke admissions pre and during COVID is of value and interest to the scientific and medical community. Although limited application as only one country- still important. Would like the authors to comment whether these individuals who didn't come to hospital- what do the authors anticipate the long-term effects of people not coming to the hospital with stroke? Was there a rise in stroke related deaths? Do the authors know this?

Authors’ response: We thank the reviewer for this important suggestion. We have identified and corrected some grammatical mistakes after careful revision of the manuscript, and further had it reviewed by a professional proof reader.

Like the reviewer we were also curious about the fate of the individuals who possibly failed to turn up into hospitals for stroke treatment during the pandemic. To our knowledge, no records of these individual were available nor any information on them including mortality were collected. Unfortunately, therefore, we are not in a position to confirm any definitive consequence of missing timely hospital treatment. However, we speculate fatalities or detrimental long-term effects including impaired speech and paralysis for these individuals.

We have now included the following statement in the Discussion section:

*“While deaths and detrimental long-term effects including impaired speech, paralysis of limbs and restricted physical abilities were speculated for missing timely hospital treatment, the extent of such consequences could not be confirmed due to unavailability of required data.”*

Reviewer #3:

Comments: While the concept is interesting, the epidemiology methods are not rigorous and the study has not been performed in a manner that will actually answer the question. Currently, total infection rates are not known, comparisons across different time periods may not be valid as SARS-CoV-2 could have been present well before March, and the pathophysiology of COVID-19 related stroke is not well known yet, so it's not clear how long after an infection that a stroke may develop.

Authors’ response: We thank the reviewer for the careful observation and comment.

In response, we first we want to make it clear that the only question investigated in this paper is whether the stroke admission data supported the general fear that there was a rise in stroke cases during the time (after March 2020), which saw a surge in COVID-19 infections in Bangladesh. The fact that an incidence of stroke requires special medical treatment available only at stroke caring facilities/hospitals it is reasonable to expect that admission data will fairly reflect major changes in the number of new stroke cases. It is evident from the data and analysis that, contrary to the fear, there is a significant drop in stroke admissions during the wide-spread outbreak of the virus compared to the earlier period.

We understand the reviewer’s concern regarding the validity of the study design for answering the research question. While we know that a prospective study following hospital admitted COVID-19 patients up to the development of any stroke would be a much better design, retrospective studies have been used to investigate similar questions (e.g., Hoyer et al., 2020). We, however, cited comparing just six months of the same year 2020 as a possible limitation of our study. We also acknowledged that comparison across the same periods of the COVID-19-year of 2020 and an earlier year, as conducted in Hoyer et al. (2020), would have been a fairer comparison. Nonetheless, we could not pursue this due to the unavailability of the required longer time series from a relatively young stroke unit and clearly pointed out this limitation in the manuscript.

We want to further argue that the retrospective comparison of admissions before and including March (our pre-COVID period) and the period thereafter (our COVID period) is a reasonable, if not ideal, comparison. First, while the reviewer is correct in pointing out the possibility of the presence of SARS-COV-2 in Bangladesh before March, we have strong reasons to believe that the total number of infections prior to March was much lower than that after March since when official statistics reported a rapid increase in the number of infections in the country.

Second, while it is true that the pathophysiology of COVID-19 related stroke is still not perfectly known, we found a fair amount of evidence (as cited in the paper) which links COVID-19 to coagulopathy causing venous and arterial thrombosis (Oxley et al., 2020, Beyrouti et al., 2020) which are major risk factors for stroke. A recent study further found significant association between the severity of COVID-19 and increased risk of acute stroke (Siepman et al., 2020).

Most importantly, although the exact duration of development of stroke in COVID-19 patients may not be definitely known, the study by Beyrouti et al. (2020) found that five out of six patients admitted with both COVID-19 and stroke actually developed ischaemic stroke within 8-24 days after COVID-19 symptom onset. Since we compared 3-month (90-day) average admissions before and after March we reasonably expected a significant number of new developments of stroke to occur during the latter period of higher rates of infection and get reflected accordingly in the admission data during that period. This made us believe that the comparisons we made are fair for testing our hypothesis. The fact that the empirical evidence goes in the opposite direction of the hypothesis only made it even more interesting.

We have already cited the references used in making our arguments in the original manuscript and included further evidence.

References:

1. Hoyer, C., Ebert, A., Huttner, H.B., Puetz, V., Kallmünzer, B., Barlinn, K., Haverkamp, C., Harloff, A., Brich, J., Platten, M. and Szabo, K., 2020. Acute Stroke in Times of the COVID-19 Pandemic: A Multicenter Study. *Stroke*, pp.STROKEAHA-120.
2. Oxley, T.J., Mocco, J., Majidi, S., Kellner, C.P., Shoirah, H., Singh, I.P., De Leacy, R.A., Shigematsu, T., Ladner, T.R., Yaeger, K.A. and Skliut, M., 2020. Large-vessel stroke as a presenting feature of Covid-19 in the young. *New England Journal of Medicine*, *382*(20), p.e60.
3. Beyrouti, R., Adams, M.E., Benjamin, L., Cohen, H., Farmer, S.F., Goh, Y.Y., Humphries, F., Jäger, H.R., Losseff, N.A., Perry, R.J. and Shah, S., 2020. Characteristics of ischaemic stroke associated with COVID-19. *Journal of Neurology, Neurosurgery & Psychiatry*.
4. Siepmann, T., Sedghi, A., Simon, E., Winzer, S., Barlinn, J., de With, K., Mirow, L., Wolz, M., Gruenewald, T., Schroettner, P. and von Bonin, S., 2020. Increased risk of acute stroke among patients with severe COVID‐19: a multicenter study and meta‐analysis. *European Journal of Neurology*.
