# Supplementary material for: Impact of COVID-19 on hospital admission of acute stroke patients in Bangladesh
Source: PLoS One. 2021 Jan 13;16(1):e0240385. doi: 10.1371/journal.pone.0240385 (PMC7806171; doi:10.1371/journal.pone.0240385)
Supplement: Attachment — Submitted filename: Response to Reviewers 21.12.2020.docx [file pone.0240385.s003.docx]

**Response to the Academic Editor and Reviewers**

**Response to the Academic Editor:**

Comment 1: In order to provide a more complete information to our readers on the topic, we would like to emphasize the importance to cross referencing very recent material on the same topic published in "PLoS ONE ".

Authors’ response: We would like to thank the academic editor for the comment. We have added relevant points in the “Introduction and Discussion” section from the following three related articles published very recently in PLos ONE. The articles are cited in text and listed in the reference according to the following serial numbers:

2. Campiglio L, Priori A. Neurological symptom in acute COVID-19 infected patients: A survey among Italian physicians. PLoS One. 2020 Sep; 15 (9): e0238159.
5. Jillella DV, Janocko NJ, Nahab F, Benameur K, Greene JG, Write WL, et al. Ischemic stroke in COVID 19: An urgent need for identification and management. PLoS One. 2020 Sep; 15 (9): e0239443.
23. Stöhr E, Aksoy A, Campbell M, Al Zaidi M, Öztürk C, Vorloeper J, et al. Hospital admissions during COVID-19 lock-down in Germany: Differences in discretionary and unavoidable cardiovascular events. PLoS One. 2020; 15 (11): e0242653.

Comment 2: Added “Cao, J., Tu, W. J., Cheng, W., Yu, L., Liu, Y. K., Hu, X., & Liu, Q. (2020). Clinical features and short-term outcomes of 102 patients with corona virus disease 2019 in Wuhan, China. *Clinical Infectious Diseases*.” in revision text.

Authors’ response: We would like to thank the academic editor for suggesting this important and timely article on clinical presentation and outcome of COVID patients. We have added a few more words in the introduction and cited the article as a reference in the revised text.

Comment 3: Resubmit figures.

Authors’ response: We have uploaded the files to PACE digital diagnostic tool to covert to appropriate tif format and made the adjustments as per the requirement of the journal.

**Response to the Reviewers’ comments:**

**Reviewer 1:**

Comment: They have tried to improve the submitted manuscript grammatically, in my humble opinion it could have improved further.

Authors’ response: We would like to thank the reviewer for the comment. We can ensure that we have tried to improve the grammar of the manuscript to the best of our abilities. We are hopeful that it now meets the required standard of the journal.

**Reviewer 2:**

Comment: No further comments for the authors of this manuscript. They addressed all of my concerns in the revised document.

Authors’ response: We thank the reviewer for his/her important comments raised previously and we are happy to learn that we managed to address all his/her concerns.

**Reviewer 3:**

Comment: Thank you for your attention to all the reviewers' comments and questions. I believe that the authors have addressed all comments to their abilities.

Authors’ response: We thank all the reviewers for their comments on the original manuscript. We would like to ensure that we tried to address each comment to the best of our abilities. We believe that the comments helped us greatly to improve the manuscript.
